# An Intranasal Vaccination with a Recombinant Outer Membrane Protein H against Haemorrhagic Septicemia in Swamp Buffaloes

**DOI:** 10.1155/2020/3548973

**Published:** 2020-05-26

**Authors:** Anucha Muenthaisong, Boondarika Nambooppha, Amarin Rittipornlertrak, Pallop Tankaew, Thanya Varinrak, Korkiat Muangthai, Kheemchompu Atthikanyaphak, Takuo Sawada, Nattawooti Sthitmatee

**Affiliations:** ^1^Department of Veterinary Biosciences and Public Health, Faculty of Veterinary Medicine, Chiang Mai University, Chiang Mai 50100, Thailand; ^2^Central Laboratory, Faculty of Veterinary Medicine, Chiang Mai University, Chiang Mai 50100, Thailand; ^3^Bureau of Veterinary Biologics, Department of Livestock Developments, Ministry of Agriculture and Cooperative, Nakhon Ratchasima 30130, Thailand; ^4^Laboratory of Veterinary Microbiology, Nippon Veterinary and Life Science University, Musashino, Tokyo 180-8602, Japan; ^5^Excellence Center in Veterinary Bioscience, Chiang Mai University, Chiang Mai 50100, Thailand

## Abstract

Hemorrhagic septicemia (HS) is an important infectious disease in cattle and buffaloes, caused by *Pasteurella multocida* B:2 and E:2. The intranasal recombinant OmpH-based vaccine was successfully used to protect dairy cattle from HS in a previous study. Thus, this study aimed to examine the protective ability of that vaccine among buffaloes. Four groups of Thai swamp buffaloes received different vaccines and were labeled as 100 or 200 *μ*g of the rOmpH with CpG-ODN2007, commercial HS bacterin vaccine, and nonvaccinated control groups. Sera and whole blood were collected to examine the antibody levels and cellular immune response using indirect ELISA and MTT assay, respectively. Challenge exposure was performed with virulent *P. multocida* strain M-1404 serotype B:2 on day 72 of the experiment. The antibody titers to *P. multocida* among immunized buffaloes were significantly higher than in the control group (*p* < 0.01), especially the 200 *μ*g of the rOmpH group. The stimulation index (SI) of the intranasally vaccinated groups revealed significantly higher levels than the nonvaccinated group (*p* < 0.01), but not different from the intramuscularly commercial HS vaccine. The clinical signs and high fever were observed after challenge exposure in the nonvaccinated group, while it was not observed among the 200 *μ*g of rOmpH immunized buffaloes. The other immunized groups showed partial protection with transient fever. In conclusion, the rOmpH-based intranasal vaccine could elicit protective ability and induce antibody- and cell-mediated immune response against virulent *P. multocida* strain among swamp buffaloes.

## 1. Introduction

Hemorrhagic septicemia (HS) is an acute and highly fatal systemic disease occurring in cattle and buffaloes in tropical regions, caused by specific serotypes of *Pasteurella multocida* B:2 (Asian serotype) and E:2 (African serotype) [1–4]. Various clinical signs have been described for HS in cattle and buffaloes, mainly the respiratory and digestive tracts [5]. Moreover, there was evidence of involving the nervous system in the pathogenesis of HS in buffaloes [6]. The outbreaks of the disease lead to economic losses in meat and milk product-related industries. In the countries that drive the economy with agriculture including Thailand, swamp buffalo is an important animal in the livestock. There are two main reasons for raising swamp buffaloes: agricultural activities and conservation. With regard to the population statistics on buffaloes for the entire country, there were a total of 1.2 million buffaloes in Thailand in 2018 [7]. HS is one of the important infectious diseases of buffaloes as buffaloes are more disease-sensitive hosts for HS than cows [4, 8]. The mortality rate among buffaloes by HS is also higher than among other ruminant species. The outbreaks of the disease lead to economic losses in meat and milk product-related industries. Therefore, the suitable prevention of HS in buffaloes is needed in this area.

Several strategies have been developed to control HS disease including vaccinations. Vaccinations of HS in animal endemic areas are the only practical approach to preventing this disease [4, 8, 9]. Various formulations of HS vaccines are available to treat animals, including inactivated vaccines, live vaccines, purified capsular extract vaccines, and combined vaccines [2]. The parenteral administration of HS is an oil-adjuvant formulation, but it is inconvenient for practical use and may induce stress in animals [10]. Although several conventional vaccine formulations are commercially available, the quest for suitable broadly protective HS vaccines with long-lasting immunity is on the upsurge [4]. Up to date, a variety of modern vaccines, including recombinant vaccines, have been developed as veterinary vaccine candidates for HS prevention [11]. The outer membrane protein H (OmpH) is a surface antigen of *P. multocida*, recognized as an immunodominant porin and potential target to be a vaccine candidate for pasteurellosis in domestic animals [10, 12–15]. Concerning the buffaloes and cattle, the 37 kDa OmpH of *P. multocida* serotype B:2 has been identified and suggested as a highly antigenic protein [16–19]. Also, there was a report that involved the development of a recombinant OmpH (rOmpH) of *P. multocida* strain M-1404 as an alternative vaccine and demonstrated a sufficient level of protection against HS among vaccinated dairy calves [10].

Mucosal vaccination via the nasal route is recognized as a noninvasive method of administration and has several advantages over traditional approaches [20]. Since the route of *P. multocida* infection in buffaloes and cattle is mainly at the upper respiratory tract, the intranasal vaccination would be suitable defense mechanisms against invading pathogens [10, 20]. The objective of this study was to formulate an appropriate concentration of rOmpH-based intranasal vaccine and determine the protective capability against *P. multocida* challenge exposure among buffaloes. Moreover, the antibody response and lymphocyte activation against the rOmpH-based intranasal vaccine were also investigated by an indirect ELISA, lymphocyte proliferation, and MTT assay.

## 2. Materials and Methods

### 2.1. *P. multocida* Strain and Culture

*P. multocida* strain M-1404 serotype B:2 was grown in the brain heart infusion broth (BHI broth; Difco Laboratories, Detroit, MI, USA) at 37°C for 6 h and was then cultured on brain heart infusion agar (BHA; Difco) at 37°C for 18 h. One single colony was selected for the preparation of bacterial suspension for challenge exposure [10]. Heat extract antigen was prepared according to the method described previously for ELISA detection [21].

### 2.2. Recombinant OmpH Production

The expression vector pQE-30 containing the *ompH* gene of *P. multocida* strain M-1404 (serovar B:2) (pQE-30/*ompH*) in *E. coli* strain M15 was constructed and obtained from a previous study [10] to produce the recombinant OmpH (rOmpH). *E. coli* strain M15 containing the pQE-30/*ompH* vector was cultured in selective LB broth containing 100 *μ*g/ml ampicillin and 25 *μ*g/ml kanamycin (Sigma Aldrich, St. Louis, MO, USA) until OD_600_ reached 0.5 and was then used to induce recombinant protein production with IPTG (isopropyl-*β*-D-thiogalactopyranoside; Takara, Otsu, Japan) at a final concentration of 1 mM. The recombinant protein was purified by an electroelution method that was described previously [10, 22]. The rOmpH concentration was measured using a BCA protein assay kit (Pierce®, Rockford, IL, USA) following the manufacturer's instructions.

The eluted rOmpH was adjusted into 100 *μ*g or 200 *μ*g of protein/0.5 ml of the intranasal rOmpH vaccine administered with an equal proportion of 10 *μ*g of cytosine-phosphate-guanosine oligodeoxynucleotides 2007 (CpG-ODN 2007; Invivogen, San Diego, CA, USA) (see the next section).

### 2.3. Experimental Animals

In this study, twenty-four of 4- to 6-month-old swamp buffaloes were divided into 4 groups according to the four types of vaccine formulations they were assigned (*n* = 6 buffaloes in each group). The groups were labeled as 100 *µ*g each rOmpH + CpG ODN (group 1), 200 *µ*g each rOmpH + CpG ODN (group 2), commercial HS vaccine (group 3), and nonvaccinated controls (group 4). The rOmpH vaccine in groups 1 and 2 were administered via intranasal route with 10 *μ*g of CpG ODN, while the commercial HS vaccine in group 3 was administered intramuscularly (Table 1). The buffaloes were housed initially in one pen with four separated rooms for each group at the Bureau of Veterinary Biologics, Department of Livestock Development, Ministry of Agriculture and Cooperatives, Pak Chong, Nakhon Ratchasima, Thailand. The buffaloes were screened for anti-*P. multocida* serovar B:2 antibody by an indirect ELISA detection assay as has been described previously [21]. Prior and subsequent to determining challenge exposure, buffaloes were housed indoors in an Animal Biosafety Level 2 isolation barn. The Institutional Animal Care and Use Committee approved of all practices that were employed in this study (approval number R19/2560).

### 2.4. Immunization and Challenge Exposure

The buffaloes were divided into four groups based on the vaccine formulations mentioned above (*n* = 6 in each group). The vaccines were administered intranasally three times at three-week intervals on days 0, 21, and 42 (groups 1 and 2) or intramuscularly twice with a one-month interval on days 0 and 30 (group 3). No vaccination was administered to the buffaloes in group 4. All buffaloes were then observed for clinical signs and behaviour changes in pre- and postimmunization stages. Sera of all buffaloes were obtained on days 0, 7, 28, 35, 49, and 63.

At day 72 of the experiment, the immunized and nonimmunized animals have been challenged with 10^3^ CFU/ml of *P. multocida*strain M-1404 via an intranasal route [10]. After challenge exposure, all buffaloes were observed for 10 days by the attending veterinarian. Veterinarian observations were made by following subjective and objective criteria using a commonly used clinical evaluation system for vital signs, depression, appetite, respiratory signs, and rectal temperature as described previously [10, 23]. At the end of the experiment, or if the experiment was terminated, buffaloes were treated with injectable Synulox™ ready-to-use injection (8.75 mg/kg; Zoetis, Florham Park, NJ, USA) for 5 days and returned to outside pens for observation for a period of 10 days. The experiment would be terminated if clinical signs of HS or the disease appeared to develop in the experimental groups.

### 2.5. ELISA Test to Evaluate Sera Antibody against *P. multocida*

The heat extract antigen of *P. multocida* strain M-1404 was prepared using the saline extraction method as has been described previously [21]. Sera were assayed for anti-*P. multocida* strain M-1404 antibody using ELISA detection against the heat extract antigen as had been described in the following passage. Microtiter plates (Nunc-Immuno Plate MaxiSorp, Intermed, Roskildes, Denmark) were coated with 160 *μ*g/ml of the heat extract antigen that had been diluted in a coating buffer. Serum dilutions for the various assays were prepared at 1 : 100 for the heat extract antigen in PBS + 1% skim milk, which were within the linear range of established dilution curves. Horseradish peroxidase-conjugated goat anti-bovine IgG (KPL, Gaithersburg, MD, USA) diluted at 1 : 2000 was used as a secondary antibody, and tetramethylbenzidine (TMB; KPL) was used as the substrate. The color reaction was stopped by adding 50 *μ*l of 3 M H_2_SO_4_. The absorbance of each well was read at a wavelength of 450 nm using an automatic plate reader (AccuReader, Metertech, Taipei, Taiwan, ROC), and the results were expressed as optical density (OD). The cut-off point of this indirect ELISA was set to 0.128 [21].

### 2.6. Lymphocyte Proliferation and MTT Assays

The preparation method of the peripheral blood mononuclear cells (PBMCs) was slightly modified as described previously [24]. Briefly, blood (10 ml) was collected in heparinized tubes from the jugular vein. Blood samples were diluted with sterile PBS (pH 7.2) to a final volume of 15 ml and then underlaid with 10 ml of Lymphoprep™ (Stemcell). Peripheral blood mononuclear cells (PBMCs) were separated as a thin layer over the Lymphoprep by centrifugation at 400 × *g* for 30 minutes at 25°C. PBMC fractions were collected and the red blood cells were lysed with the 1× RBC lysis buffer for 5 minutes at 37°C. They were washed twice with RPMI-1640 medium by centrifugation at 700 × *g* for 7 minutes at 25°C. Cell pellets were then resuspended with 2 ml of RPMI-1640 medium supplemented with antibiotics-antimycotics (Invitrogen) and 10% fetal calf serum (FCS) (Invitrogen) before enumerating the number of cells.

PBMCs at 2 × 10^5^ cells/well were transferred into a 96-well microtiter plate in triplicate and stimulated with 5.0 *μ*g/ml at a final concentration of the heat extract antigen of M-1404. The culture medium and 10 *μ*g/ml of ConA (ConcanavalinA, C-2010, Sigma) were used as the control. Cells were incubated for 48 h at 37°C in an atmosphere containing 5% CO_2_. The lymphocyte proliferation was determined using 3-(4,5-di-methylthiazolyl-2)-2,5-diphenyltetrazolium bromide (MTT) assay, which was based on the cleavage of a tetrazolium salt by mitochondrial dehydrogenases of the viable cells [25]. After 48 hours of incubation, 10 *μ*l of 12 mM MTT solution (Sigma-Aldrich) was added to each well, and the plates were incubated for 3 hours at 37°C. After 3 hours of incubation, 100 *μ*l of 10% SDS containing 0.01 M HCl was added and incubated for 3 hours at 37°C. The optical density (OD) was measured by reading the absorbance at 540 nm using an automatic plate reader (AccuReader). The stimulation index (SI) value was calculated by equivalent to the mean absorbance of stimulated wells/mean absorbance in the media wells.

### 2.7. Data Analysis

Statistical analyses of the antibody titers and SI value between the vaccinated groups and the nonvaccinated control group were performed using a repeated-measures ANOVA test. The level of significance was recorded at *p* < 0.05.

## 3. Results

### 3.1. Immunization and *P. multocida* Challenge Exposure

After challenged with *P. multocida* strain M-1404, all experimental animals have been observed the clinical signs and measured the rectal temperature within a period of 24 hours. All buffaloes in group 4 displayed high rectal temperatures (104.0–105.0°F) with clinical signs including dullness, reluctance to move depression, and anorexia at 4 to 6 hours after being challenged by the intranasal route. One hundred percent of the morbidity rate in group 4 challenged with *P. multocida* strain M-1404 was reported within 24 h. Subsequently, the experiment in group 4 was terminated due to continually rectal temperature. On the other hand, the protective levels of 66.67%, 100%, and 83.33% were provided by 100 *μ*g rOmpH, 200 *μ*g rOmpH, and commercial HS vaccine groups, respectively, by showing a normal range of body temperature (99.0–102.0°F) with no clinical signs of HS (Table 1). However, some of the buffaloes in those groups manifested transient fever (103–103.5°F) but returned to a normal temperature on day 2 after challenge exposure.

### 3.2. Determination of Antibody Titers against HS Vaccination

**Figure 1:**
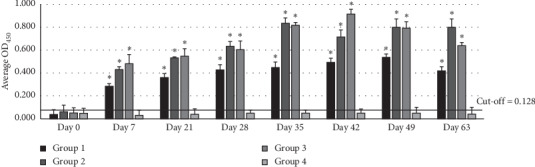
Serum IgG profile of buffalo immunized with different vaccine formulations against rOmpH of *P. multocida* strain M-1404 by indirect ELISA. Asterisk (∗) represents the significant differentiation of antibody IgG level compared to the nonvaccinated control group (*p* < 0.01).

The levels of antibody titers among Thai swamp buffalos are shown in Figure 1. The levels of antibody titers of swamp buffaloes against the heat extract antigen on day 0 indicated a low cut-off value of 0.128. As observed, the average antibody levels of buffaloes in groups 1, 2, and 3 were empirically elevated on day 7 after having been given the vaccination. However, the nonimmunized buffaloes of group 4 were found to be seronegative to HS, as the average antibody levels throughout the experimental period were lower than the cut-off value. The average antibody titer levels of buffaloes in groups 1, 2, and 3 were continually elevated and significantly higher than the average antibody level of group 4 (*p* < 0.01). Overview, a peak of antibody titer in all groups was found 2 weeks after initial and booster immunization. With regard to the route of vaccine administration, the average antibody titer levels of group 2 (200 *μ*g of rOmpH, intranasal route) were not different from the levels of the buffaloes in group 3 (commercial HS vaccine, intramuscular route) throughout the experimental.

### 3.3. Cellular Immune Response Using MTT Assay

**Figure 2:**
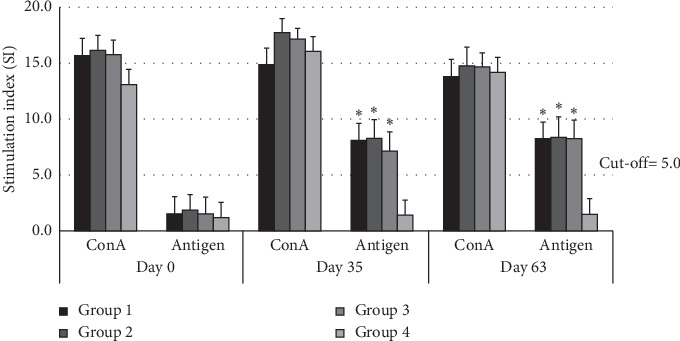
Stimulation index (SI) of buffalo PBMCs immunized with different vaccine formulations against Con A and rOmpH of *P*. *multocida* strain M-1404 antigen. Asterisk (∗) represents the significant differentiation of SI value compared to the nonvaccinated control group (*p* < 0.01).

The cellular immune responses in buffaloes are shown in Figure 2. The cut-off values of the stimulation index were designated into 5 units, and the cellular immune responses against an antigen of *P. multocida* on day 0 were lower than the cut-off value in all groups. The responses of groups 1, 2, and 3 increased, and the stimulation index values were higher than the cut-off value on days 35 and 63 of the experiment. The stimulation index values of the buffaloes in groups 1, 2, and 3 were significantly higher than those of the buffaloes in group 4 against an antigen (*p* < 0.01).

## 4. Discussion

*P. multocida* is a Gram-negative bacterium that plays a role in multihost diseases [26]. It causes haemorrhagic septicemia, a disease normally found in Asia, Africa, and Europe in cattle, buffaloes, and bison [27]. HS occurring in buffaloes and cattle is caused by one of two specific serotypes of *Pasteurella multocida* B:2 and E:2 [2, 4]. The clinical signs and lesions of HS in cattle and buffaloes were described mainly in the respiratory and digestive tracts due to bacterial colonization that takes place in the respiratory system [5]. The outbreaks of the disease provided serious economic losses. Therefore, several strategies have been employed to control and protect these animals against HS including vaccination [4, 8]. Currently, the HS vaccines being used involve either inactivated bacteria or live-attenuated bacteria [2, 4]. Bacterin vaccines are inexpensive to produce, but they often cause side effects and provide very limited protection against heterologous serotype infections. Notably, the high body temperature was reported in conventional routes after intramuscular vaccination [16]. The mucosal vaccine could be used as an alternative route for vaccination against HS in cattle due to the first line of defense against infection of *P. multocida* at the mucosal surface [10]. This present study provided the possibility of intranasal vaccination with a recombinant OmpH protein from *P. multocida* against HS in swamp buffaloes with strong antibody response and clinical protection levels.

The outer membrane protein H (OmpH) is a major outer membrane protein in an envelope of *P. multocida*. The OmpH of *P. multocida* serovar B:2 was identified as 37 kDa and acknowledged as an appropriate candidate for use as a vaccine against HS [2, 18]. A previous study has reported on the rOmpH-based intranasal vaccine and revealed a protective capability in dairy calves [10]. An intranasal vaccine induced both the serum IgG and secretory IgA levels that were significantly higher than the parenteral vaccine and reduced clinical signs after *P. multocida* challenge exposure [10]. However, there have not been any reports on achieving protection of the rOmpH protein vaccine that had been isolated from *P. multocida* in buffalo, a disease-sensitive host for HS. Therefore, the intranasal rOmpH protein vaccine was developed and applied in buffaloes in this study. This could allow researchers and veterinarians to replace the conventional vaccine and use it in the field as an alternative vaccine presently being used.

Antibodies of the IgG isotype play an essential role against pathogenic microorganisms and revealing the humoral immune responses [28, 29]. With regard to our results, the antibody titer profiles in this study were similar to those in a previous study using the rOmpH-based intranasal vaccine against *P. multocida* challenge exposure in dairy calves [10]. The antibody titer level was rapidly increasing within 2 weeks after booster. Regarding the formulation of the vaccine, the concentration of 100 *μ*g rOmpH in the intranasal vaccine provided a lower average antibody level than the vaccine containing 200 *μ*g rOmpH, indicating that the rOmpH-based intranasal vaccine formulated in this study is dose dependent. In comparison to the previous study, the efficient concentration of rOmpH for dairy calves was found to be 50 or 100 *μ*g per vaccine dosage [10]. This can be explained by observing how the host species reacted in terms of an immune response during the postvaccination stage of administering the vaccine. In the present study, the CpG ODN was incorporated into the intranasal vaccine because CpG is considered to be a suitable adjuvant in intranasal for protein antigens [30]. The CpG ODN adjuvant has been found to induce Th1 humoral response and increase production of antigen-specific IgG, IFN-*γ*, and IL-2 via TLR9 [31, 32]. A previous study demonstrated the development of recombinant OmpH protein of *P. multocida* strain A:3 with CpG ODN vaccine formulation can strongly induce IgG and serum IFN-*γ* in mice [32]. It is a possible explanation for high IgG levels and no significant different antibody titer induced by 200 *μ*g of the OmpH intranasal vaccine when compared to the intramuscular bacterin vaccine. Contrastingly, the *P. multocida* live-attenuated vaccine from the previous study showed significantly increased IgG antibody concentrations after the second vaccination in intramuscular but not in intranasal vaccinated calves [16]. Consequently, the results in this study support the use of OmpH as a potential immunogen with CpG ODN developing intranasal vaccination to produce sera IgG against *P. multocida* in buffaloes.

Clinical signs and rectal temperature monitoring were performed as a parameter to observe HS development. Remarkably, the clinical protection levels in nonimmunized and immunized buffaloes in this study were correlated to the antibody IgG levels. The results showed high protection levels in the buffaloes in groups 2 and 3 which displayed the significantly elevated of the antibody levels, were protected 100% (6/6) and 83.33% (5/6), respectively. However, nonimmunized buffaloes showed one hundred percent morbidity rate after challenged with *P. multocida* strain M-1404 with continually high fever, depression, anorexia, and loss of appetite, related to nonincreased antibodies, and SI index. The veterinarian decided to terminate the experiment for this nonimmunized group. The bacterial culture was obtained from the body exudate sample to ensure that an appropriate treatment strategy was employed after the termination of the experiment. Since the antibodies play a major role in neutralizing viruses and bacterial toxins [33], the results in this study support that the elevated antibody levels recorded at the postvaccination stage might be an advantage in the host's defense mechanism by reducing the clinical sign expression after challenge exposure.

Apart from the humoral immune response, the cellular immune response was also investigated. The MTT assays were used to measure viability, proliferation, and activation of cells [34, 35]. There are studies that involved a cellular immune response to OmpH of *P. multocida* in dairy calves and mice, showing a strong cellular response to the immunogen [10, 36]. The results from the MTT assay indicated that the lymphocyte activation involved in vaccination against HS, following the vaccinated buffaloes in groups 1, 2, and 3 (both intranasal rOmpH and intramuscular bacterin HS vaccines) showed significantly higher than the control group. This phenomenon indicated that cellular immunity responds to the presence of an immunogen in the vaccine and continually developed as a group of memory cells. However, the assays were unable to predict the degree of protection capability in in vivo conditions. Dose differences or the route of vaccine administrations did not show significant differences in cellular immune response using the MTT assay.

Collectively, 200 *μ*g of rOmpH plus CpG ODN formulation in this study showed the high level of antibody titer, SI index, and clinical protection level. This evidence supports the protective ability of the intranasal vaccine containing 200 *μ*g of rOmpH that could be the suitable alternative vaccine against HS in buffaloes in the veterinary field instead of the conventional vaccines.

## 5. Conclusion

This present study provided a preliminary experiment and understanding of the immune response of buffaloes immunized with the rOmpH-based intranasal vaccine against *P. multocida* strain serotype B:2 infection. In accordance with our investigation, the rOmpH-based intranasal vaccine was able to induce an antibody response and cellular response, especially a concentration of 200 *μ*g of rOmpH. Furthermore, immunized buffaloes showed reduction in the clinical signs of the disease after challenge exposure with the bacterial strain. The data revealed that the rOmpH-based intranasal vaccine could be an interesting alternative vaccine to protect the buffaloes against HS disease with improved efficacy and safety of the vaccines that are presently being used. In order to fully study the abilities of the rOmpH-based intranasal vaccine, the variety of formulations, dosages, adjuvants, and vaccine frequencies are needed for further investigation.

## Figures and Tables

**Table 1:** Vaccine formulations, number of immunizations, and protection in buffaloes.

Group	Vaccine formulations	No. of immunizations	Inoculum strain^c^	No. of buffaloes without clinical sign/total (%)
1	100 *µ*g each rOmpH + CpG ODN 10 *µ*g^a^	6	M-1404	4/6 (66.67)
2	200 *µ*g each rOmpH + CpG ODN 10 *µ*g^a^	6		6/6 (100)
3	Commercial haemorrhagic septicemia vaccine^b^	6		5/6 (83.33)
4	No vaccination	6		0/6 (0)^d^

^a^Intranasal administration with 1.0 ml/dose. ^b^Intramuscular administration with 1.0 ml/dose. ^c^Challenge inoculum was 1 ml of PBS containing approximately 10^3^ CFU/ml. ^d^This group was terminated due to the clinical signs of buffaloes.

## Data Availability

The data used to support the findings can be obtained from the corresponding author upon a reasonable request.

## References

[1] Wijewardana T. G., Wilson C. F., Gilmour N. J. L., Poxton I. R. (1990). Production of mouse monoclonal antibodies to *Pasteurella multocida* type A and the immunological properties of a protective anti-lipopolysaccharide antibody. *Journal of Medical Microbiology*.

[2] Verma R., Jaiswal T. N. (1998). Haemorrhagic septicaemia vaccines. *Vaccine*.

[3] Dabo S. M., Taylor J. D., Confer A. W. (2007). Pasteurella multocidaand bovine respiratory disease. *Animal Health Research Reviews*.

[4] Shivachandra S. B., Viswas K. N., Kumar A. A. (2011). A review of hemorrhagic septicemia in cattle and buffalo. *Animal Health Research Reviews*.

[5] Carter G. R., De Alwis M. C. L., Adlam C., Rutter J. M. (1989). Haemorrhagic septicaemia. *Pasteurella and Pasteurellosis*.

[6] Marza A. D., Jesse F. F. A., Ahmed I. M. (2016). Involvement of the nervous system following experimental infection with *Pasteurella multocida* B:2 in buffalo (*Bubalus bubalis*): a clinicopathological study. *Microbial Pathogenesis*.

[7] Office of Agricultural Economics (2018). *Ministry of Agriculture and Cooperative*.

[8] Singh V. P., Office International des Epizooties (2012). Manual of diagnostic tests and vaccines for terrestial animals. *Haemorrhagic Septicaemia*.

[9] Benkirane A., De Alwis M. C. L. (2012). Haemorrhagic septicaemia, its significance, prevention and control in Asia. *Veterinarni Medicina*.

[10] Muangthai K., Tankaew P., Varinrak T. (2018). Intranasal immunization with a recombinant outer membrane protein H based Haemorrhagic septicemia vaccine in dairy calves. *Journal of Veterinary Medical Science*.

[11] Shams H. (2005). Recent developments in veterinary vaccinology. *The Veterinary Journal*.

[12] Sthitmatee N., Numee S., Kawamoto E. (2008). Protection of chickens from fowl cholera by vaccination with recombinant adhesive protein of *Pasteurella multocida*. *Vaccine*.

[13] Thanasarasakulpong A., Poolperm P., Tankaew P., Sawada T., Sthitmatee N. (2015). Protectivity conferred by immunization with intranasal recombinant outer membrane protein H from. *Journal of Veterinary Medical Science*.

[14] Varinrak T., Poolperm P., Sawada T., Sthitmatee N. (2017). Cross-protection conferred by immunization with an rOmpH-based intranasal fowl cholera vaccine. *Avian Pathology*.

[15] Poolperm P., Apinda N., Kataoka Y. (2018). Protection against *Pasteurella multocida* conferred by an intranasal fowl cholera vaccine in Khaki Campbell ducks. *Japanese Journal of Veterinary Research*.

[16] Hodgson J. C., Finucane A., Dagleish M. P., Ataei S., Parton R., Coote J. G. (2005). Efficacy of vaccination of calves against hemorrhagic septicemia with a live *aroA* derivative of *Pasteurella multocida* B:2 by two different routes of administration. *Infection and Immunity*.

[17] Lee J., Kim Y. B., Kwon M. (2007). Outer membrane protein H for protective immunity against *Pasteurella multocida*. *Journal of Microbiology*.

[18] Hatfaludi T., Al-Hasani K., Boyce J. D., Adler B. (2010). Outer membrane proteins of *Pasteurella multocida*. *Veterinary Microbiology*.

[19] Tan H. Y., Nagoor N. H., Sekaran S. D. (2010). Cloning, expression and protective capacity of 37 kDa outer membrane protein gene (ompH) of *Pasteurella multocida* serotype B:2. *Tropical Biomedicine*.

[20] Kharb S., Charan S. (2011). Mucosal immunization provides better protection than subcutaneous immunization against *Pasteurella multocida* (B:2) in mice preimmunized with the outer membrane proteins. *Veterinary Research Communications*.

[21] Tankaew P., Srisawat W., Singhla T. (2018). Comparison of two indirect ELISA coating antigens for the detection of dairy cow antibodies against *Pasteurella multocida*. *Journal of Microbiological Methods*.

[22] Thanasarasakulpong A., Poolperm P., Tangjitjaroen W. (2016). Comparison of the effect of two purification methods on the immunogenicity of recombinant outer membrane protein H of *Pasteurella multocida* serovar A:1. *Veterinary Medicine International*.

[23] American Dairy Science Association (2010). *Guide for the Care and Use of Agricultural Animals in Research and Teaching*.

[24] Böyum A. (1968). Isolation of leucocytes from human blood. Further observations. Methylcellulose, dextran, and ficoll as erythrocyte aggregating agents. *Scandinavian Journal of Clinical and Laboratory Investigation. Supplementum*.

[25] Lignitto L., Da Dalt L., Balzan S. (2007). Use of bovine lymphocytes to assess the immunomodulatory effect of natural extracts. *Italian Journal of Animal Science*.

[26] Wilkie I. W., Harper M., Boyce J. D., Adler B. (2012). *Pasteurella multocida*: diseases and pathogenesis. *Current Topics in Microbiology and Immunology*.

[27] Jamali H., Rezagholipour M., Fallah S. (2014). Prevalence, characterization and antibiotic resistance of *Pasteurella multocida* isolated from bovine respiratory infection. *Veterinary Journal*.

[28] Twigg H. L. (2005). Humoral immune defense (antibodies). *Proceedings of the American Thoracic Society*.

[29] Nimmerjahn F. (2014). Molecular and cellular pathways of immunoglobulin G activity in vivo. *International Scholarly Research Notices*.

[30] Wang S., Liu H., Zhang X., Qian F. (2015). Intranasal and oral vaccination with protein-based antigens: advantages, challenges and formulation strategies. *Protein & Cell*.

[31] Dennis K. M., Currie D., Gursel I., Verthelyi D. (2004). Use of CpG oligodeoxynucleotides as immune adjuvants. *Immunological Reviews*.

[32] Sezer O., Özcengiz E., Gürsel I., Özcengiz G. (2012). Immunogenicity and protective efficacy of the recombinant Pasteurella lipoprotein E and outer membrane protein H from Pasteurella multocida A: 3 in mice. *Research in Veterinary Science*.

[33] Taylor P. W. (1983). Bactericidal and bacteriolytic activity of serum against gram-negative bacteria. *Microbiological Review*.

[34] Mosmann T. (1983). Rapid colorimetric assay for cellular growth and survival: application to proliferation and cytotoxicity assays. *Journal of Immunological Methods*.

[35] Boncler M., Różalski M., Krajewska U., Podsędek A., Watala C. (2014). Comparison of PrestoBlue and MTT assays of cellular viability in the assessment of anti-proliferative effects of plant extracts on human endothelial cells. *Journal of Pharmacological and Toxicological Methods*.

[36] Kumar B., Chaturvedi V. K., Somrajan S. R. (2011). Comparative immune response of purified native OmpH protein derived from *Pasteurella multocida* P52 and oil adjuvant vaccine against hemorrhagic septicemia in mice. *Indian Journal of Animal Sciences Indian*.

